# Oxidative stress and plasma ceramides in broiler chickens

**DOI:** 10.3389/fphys.2024.1411332

**Published:** 2024-07-15

**Authors:** Hillar Klandorf, Vincent Dartigue

**Affiliations:** Division of Animal and Nutritional Sciences, West Virginia University, Morgantown, WV, United States

**Keywords:** sphingolipid ceramides, oxidative stress, lipidomic approach, broiler chickens, allopurinol

## Abstract

The selection for rapid growth in chickens has rendered meat-type (broiler) chickens susceptible to develop metabolic syndrome and thus inflammation. The sphingolipid ceramide has been linked as a marker of oxidative stress in mammals, however, the relationship between sphingolipid ceramide supply and oxidative stress in broiler chickens has not been investigated. Therefore, we employed a lipidomic approach to investigate the changes in circulating sphingolipid ceramides in context of allopurinol-induced oxidative stress in birds. Day zero hatched chicks (n = 60) were equally divided into six groups; an unsupplemented control, an allopurinol group (25 mg/kg body weight), a conjugated linoleic acid (CLA) group where half of the oil used in the control diet was substituted for a CLA oil mixture, a CLA and an allopurinol group utilizing the same dose of CLA and allopurinol, a berberine (BRB) group consisting of berberine supplementation (200 mg/kg feed), and a BRB and allopurinol group, utilizing the same dose of BRB and allopurinol. Conjugated linoleic acid and berberine were utilized to potentially enhance antioxidant activity and suppress the oxidative stress induced by allopurinol treatment. Body weight, plasma uric acid, nonesterified fatty acids (NEFA) and sphingolipid ceramides were quantified. Allopurinol induced an inflammatory state as measured by a significant reduction in plasma uric acid - an antioxidant in birds as well as a metabolic waste product. Results showed that both total and saturated sphingolipid ceramides declined (*p* < 0.05) with age in unsupplemented chicks, although plasma ceramides C16:0 and 18:0 increased in concentration over the study period. Simple total and saturated sphingolipid ceremide’s were further decreased (*p* < 0.05) with allopurinol supplementation, however, this may be an indirect consequence of inducing an inflammatory state. Neither CLA or BRB were able to significantly attenuate the decline. The administration of allopurinol specifically targets the liver which in birds, is the primary organ for fatty acids synthesis. For this reason, sphingolipid ceramide production might have been unwittingly affected by the addition of allopurinol.

## Introduction

Compared to mammals, birds have a higher metabolism, elevated blood glucose concentrations and body temperatures as well as double-folded mitochondria. In addition, they also have lower concentrations of insulin compared mammals (Whittow, 1999). Despite an increase in potency of chicken insulin compared to porcine (Simon, 1989), studies in birds have also revealed that the concentration of insulin receptors varies by tissue, with muscle expressing the lowest concentration (Simon et al., 1986). These observations suggest that birds are in a constant state of insulin resistance (Simon and Rosselin, 1978). Birds, however, do not exhibit the classic symptoms associated with hyperglycemia observed in mammals, partly due to their elevated plasma uric acid concentrations, a potent antioxidant (Ames et al., 1981). Previous studies in poultry have established that allopurinol (an antagonist of xanthine and hypoxanthine in the purine degradation pathway, which blocks uric acid production) administration induces a redox state with an increase in inflammation associated with greater abundance of reactive oxygen species (Simoyi et al., 2003; Settle et al., 2015) and proinflammatory factors (Machın et al., 2004). The severity of the redox state can be adjusted by increasing dosage of allopurinol administrated (Simoyi et al., 2003). This supplementation of allopurinol in broiler chickens induces an “inflammatory state” without a “specific disease” which effectively creates a model in which to study diseases in which inflammation is predominant (e.g., Diabetes, atherosclerosis, neuropathy).

Ceramides (Cer) are sphingolipids composed of a sphingosine back bone attached to a fatty acid and considered to be inert component of waxy lipids (Wertz and Downing, 1983). The acyl moiety attached to the sphingosine back bone can vary in length (16–26 carbons), with the fatty acid attached dictated by the ceramide synthase homologues (1–6) involved (Andrieu-Abadie et al., 2001; Bikman and Summers, 2011). However, in recent years, ceramides have been ascribed a variety of metabolic effects, most notably a reliable biomarker of oxidative stress and neurodegenerative diseases in mammals (Cutler et al., 2002; Cutler et al., 2004). In humans, Cutler et al. (Cutler et al., 2004) linked the death of motor-neurons in amyotrophic lateral sclerosis (ALS) with an increase in ceramide accumulation in the membrane wall, which renders the cell increasingly vulnerable to oxidative stress. In dairy cows, Rico et al., 2015 (Rico et al., 2015) established a relationship between elevated plasma ceramides (Cer C20:0 and C24:0) and prolonged insulin resistance in peripartal dairy cows. If left unchecked, the ceramides downregulated protein kinase B (PKB; but also known as Akt), a second messenger in the insulin pathway, which placed the cow at increased risk of developing metabolic diseases (Jeẑek and Garlid, 1998; Haus et al., 2009; Rico et al., 2015). Due to previous application in livestock, the measurement of ceramides as an early biomarker of oxidative stress could thus be beneficial to the poultry industry.

In the current study, we measured changes in plasma ceramides as potential markers of oxidative stress after allopurinol administration as well as the ability of two compounds, conjugated linoleic acid (CLA) and berberine (BRB), to increase antioxidant (AOx) activity/expression in response to the pharmacologically induced inflammatory state. Conjugated linoleic acid, a family of 18 carbon unsaturated fatty acids, is involved in the neutralization of ROS via the uncoupling of the oxidative phosphorylation complex in the mitochondrial electron transport chain (ETC) by uncoupling protein (UCP) (Chung et al., 2005). Uncoupling protein is a transmembrane protein located in the inner mitochondrial membrane (Summers et al., 1998). Previous research has proposed a mechanism by which unsaturated fatty acids (C14 to C18) enhance UCP activity and expression. Overall, UCP raises the proton gradient in the inner membranes, which increases the probability of any unpaired electrons to react with a proton, which thus limits the formation of oxygen radicals. The second compound, berberine, with exogenous antioxidant activity, is an alkaloid compound isolated from plant root extract. Berberine possesses several benzene rings, which allows the captured electron to circulate within the conjugated double bonds (Liu et al., 2015). In addition to functioning as an exogenous Aox, BRB has been proposed to increase endogenous antioxidant (SOD1) expression and exert anti-inflammatory properties (Dartigue et al., 2021).

In view of the rapid growth rate of broilers, we hypothesized that 1) sphingolipid ceramide concentration should increase in broiler chickens during a production cycle 2) and that the addition of allopurinol should further increase this production. 3) Compounds which exhibit antioxidants properties, such as conjugated linoleic acid and berberine, were predicted to reduce the redox state induced by allopurinol (Klandorf et al., 2001; Settle et al., 2012) and therefore decrease or lower the concentration of ceramides as compared to the allopurinol supplemented group.

## Materials and methods

All procedures and experiments were approved by West Virginia University’s Institution Animal Care and Use Committee (ACUC #15-0301.1).

### Experimental design

Sixty mixed sex day old Cobb/Cobb 500 broiler chicks were obtained from Pilgrim’s Pride, Moorefield, WV. Chicks were housed within pens and maintained under a 12-h light-dark cycle with the temperature set at 27°C. One week after hatch, chicks were divided into six groups and tagged with a leg band. The broiler chicks were maintained on a starter diet (Dartigue et al., 2021) for the first 4 weeks before being placed on their respective treatments for 6 weeks (see Table 1 in Dartigue et al. (2021) for the basal diet composition): a control group (Group 1); Group 2, a conjugated linoleic acid (CLA) group in which half of the oil used in the regular diet was substituted for a mixture of 50/50 *cis*-9 *trans*-11 and *trans*-10 *cis*-12 isomers CLA oil (0.80% CLA oil diet) (Klandorf et al., 2001); Group 3 (BRB) was supplemented with berberine (200 mg/kg of feed) (Settle et al., 2012); Group 4 (ALLO) the diet was supplemented with allopurinol (25 mg/kg BW) (Lightle et al., 2000); Group 5 (CLA + ALLO) and Group 6 (BRB + ALLO) mimicked the CLA and BRB treatments with the addition of allopurinol (25 mg/kg BW) for 2 weeks to induce a state of oxidative stress (Klandorf et al., 2001; Simoyi et al., 2003).

Berberine (chloride hydrate, purity >99%) and allopurinol were purchased from Sigma-Aldrich (Saint Louis, MO, United States). The CLA was purchased commercially (Vitamorph Labs, Highland, IN, United States) as 1200 mg capsules. Food and water were provided *ad libitum*.

### Sampling procedure

After 2 weeks on treatment, blood samples (4 mL) were obtained from six birds per pen from the brachial (wing) vein. The same birds were sampled weekly for the next three subsequent bleedings: for a total of four bleedings. Each blood sample was placed in a heparinized tube and stored on ice. The blood samples were centrifuged at 2000rpm for 20 min at 4°C. Plasma was then transferred to micro-centrifuge tube and stored in a −80°C freezer pending analyses.

### Sample analysis

Plasma free fatty acids concentration was measured using an enzymatic colorimetric method (HR Series NEFA-HR, Wako chemicals United States Inc., Richmond VA). Plasma uric acid (PUA) was measured using *Infinity* ^*™*^ *Uric Acid Liquid Stable Reagent* assay from Thermo Scientific (Waltham, MA, United States) (Lightle et al., 2000). Ceramides were extracted using a modified Bligh and Dyer with a C12:0 Ceramide internal standard (Avanti Polar Lipids, Alabaster, AL). Extraction and measurement of ceramide were as described in Rico et al. (Rico et al., 2015). Concentrations of plasma ceramides C16:0, 18:0, 20:0, 22:0 and 24:0 were measured.

### Statistical analysis

The statistical program Statistical Analysis Software (version 9.3; SAS Institute Inc., Cary, NC) was used to analyze the data. Both time and treatment effects were analyzed. The response variables (PUA, Ceramides, bodyweight) were analyzed using a mixed effects model in which time (study week) and treatment were included as fixed effects while individual was included as a random effect to account for the repeated measures design. A measure of dependence in both time and treatment effects was performed using a Pearson’s contingency coefficient. Values are expressed as least square (LSM) ± standard error (SE) unless stated otherwise. Statistical significance was set at *p <* 0.05 and 0.01.

## Results

**FIGURE 1:**
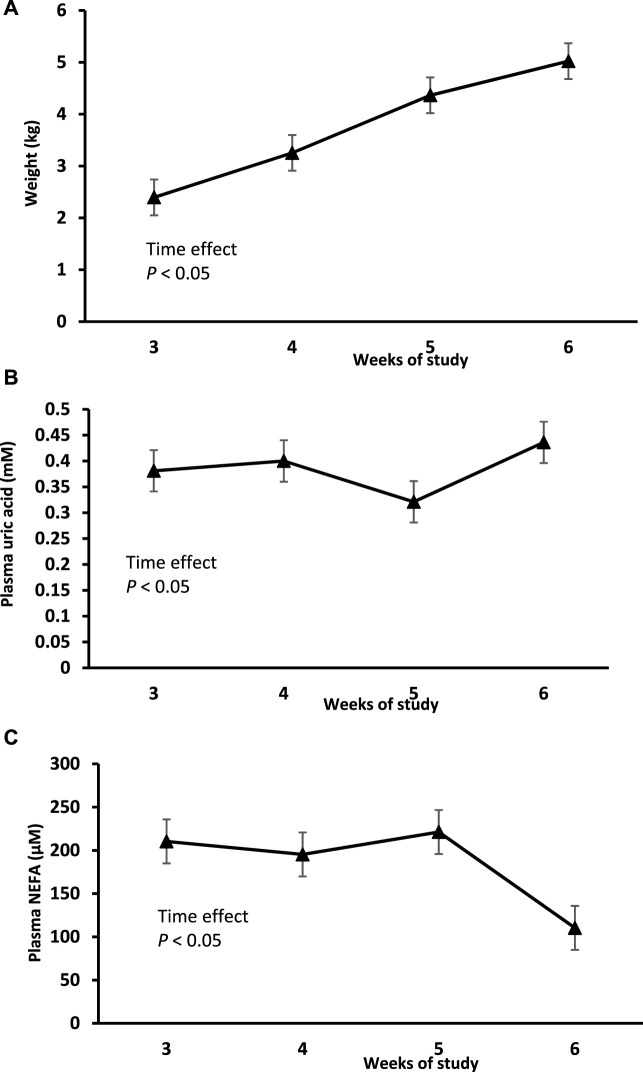
Changes in body weight **(A)**, plasma NEFA **(C)** and plasma uric acid **(B)** over time in controls chicks. Values are expressed as means ± standard error. The notation a represents significance compared to control whereas b compares to the ALLO group (*p* < 0.05).

There was a significant increase in body weight (BW) in controls over the 4 weeks of the sampling period (Figure 1A). Plasma uric acid concentrations in controls remained relatively constant around 0.4 mM (Figure 1B). In controls, NEFA remained level around 200 μM during weeks 3–5 of the study before significantly (*p* < 0.05) declining to 100 μM (Figure 1C).

**FIGURE 2:**
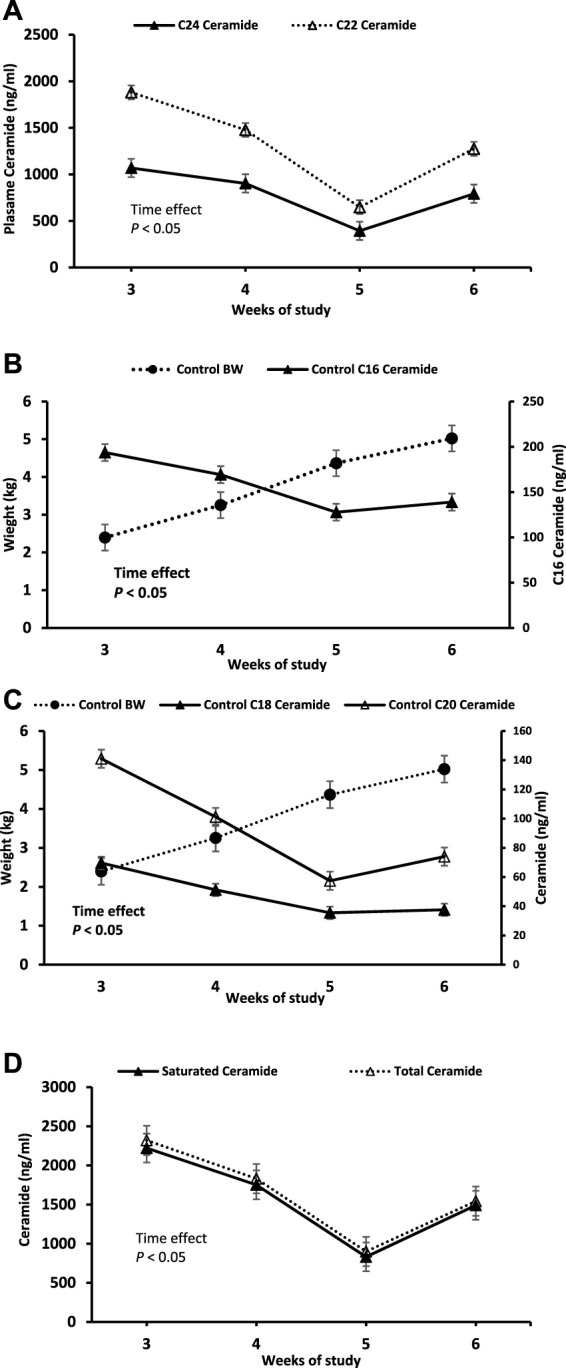
Change in plasma ceramides C22:0, C24:0 **(A)**, bodyweight and ceramides C16:0 **(B)**, body weight and ceramides C18:0 and C20:0 **(C)**, Total and saturated ceramides **(D)** over time. Values are expressed as means ± standard error.

Plasma ceramides C22 and C24 were observed to have the highest initial concentrations at ∼–1800 and 1000 ng/mL, respectively (Figure 2A). These were followed by plasma ceramide C16 with a concentration around 200 ng/mL (Figure 2B). Plasma ceramides C18 and C20 were the least expressed with concentrations ranging from 30 to 140 ng/mL (Figure 2C). The different species of plasma ceramide followed the same decreasing trend throughout the 4-week study period (Figure 2). Plasma Cer C16, C18, C20, C22, C24, as well as total saturated and total ceramides decreased (*p* < 0.05) between weeks 3 and 5. However, at week 6, Ceramides C20, C22, C24, total saturated and total ceramides had significantly increased compared to weeks 3–5, restoring approximately 60% of the original values (Figures 2A, C, D). There were no significant changes was observed in ceramide C16 and C18 between weeks 5 and 6 (Figures 2B, C). Notably, ceramides C16, C18 and C20 concentrations were negatively correlated (*r = -0.57*) with BW over time.

**FIGURE 3:**
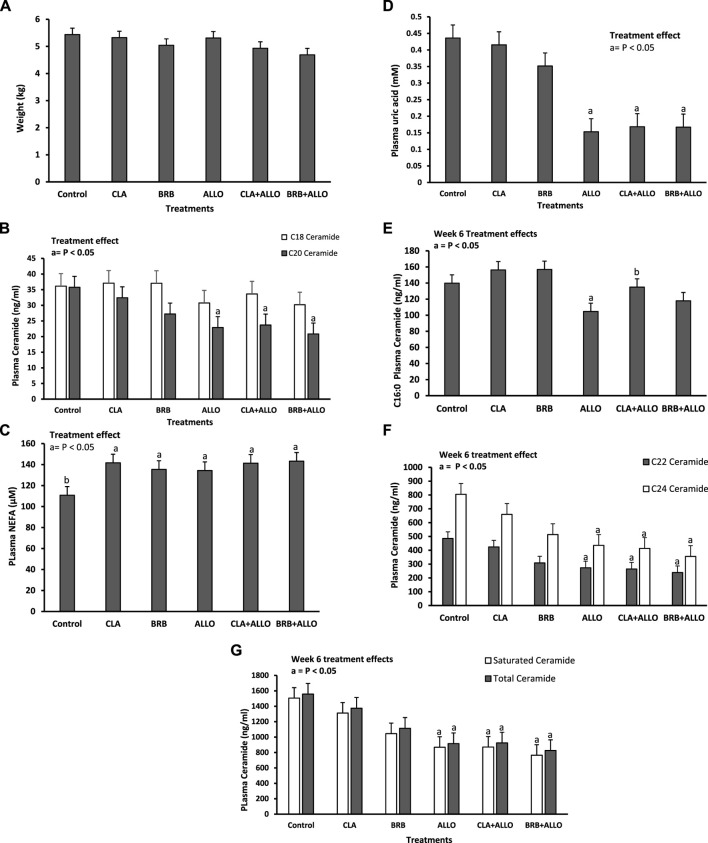
Body weight **(A)**, plasma concentrations of **(B,E,F,G)** ceramide **(D)** uric acid and **(C)** NEFA, in broiler chickens at week 6 of the study. Treatment effects of control conjugated linoleic acid (CLA), berberine (BRB), allopurinol (ALLO), conjugated linoleic acid + allopurinol (CLA + ALLO) and berberine + allopurinol (BRB + ALLO) are also shown. Values are expressed as means ± standard error. The notation a represents significance compared to control whereas b compares to the ALLO group. *p* < 0.05.

There were no treatment effects on BW (Figure 3A), which was comparable to plasma ceramide C18 concentration albeit, numerical differences were noted in the redox groups using ALLO (Figure 3B). NEFA measurements revealed a significant (*p* < 0.05) increase in concentrations in all treatments compared to controls (Figure 3C). Plasma uric acid was significantly decreased in the allopurinol supplemented groups compared to controls (Figure 3D). There was a significant decrease in the plasma C16 ALLO group while only numerical decreases were measured in the CLA + ALLO and BRB + ALLO groups compared to controls. Of note, the CLA + ALLO group was significantly higher than the ALLO group (Figure 3E). Plasma ceramide species C20, C22, C24, saturated and total ceramides decreased significantly (*p* < 0.05) in the allopurinol supplemented groups compared to controls (Figures 3B, F, G).

A positive correlation (*r = 0.50*) between PUA and plasma ceramides C22, C24 and total ceramides behavior was also measured (data not shown).

## Discussion and Conclusions

Ceramides are sphingolipids that have been positively associated with oxidative stress and metabolic diseases in mammals (Cutler et al., 2002; Cutler et al., 2004) but their role in birds has not been established. A redox state in birds can be induced by a variety of factors, amongst them allopurinol (Simoyi et al., 2003; Settle et al., 2015) with a shift towards prooxidants which can be detrimental. Previous research has demonstrated that allopurinol can reduce liver uric acid production by inhibiting liver xanthine oxidase (Simoyi et al., 2003; Settle et al., 2012). The decrease in PUA, a potent antioxidant in birds, can lead to state of oxidative stress characterized by overall inflammation but also liver inflammation (Settle et al., 2012; Settle et al., 2015). In addition, Settle et al. (Settle et al., 2012) determined that various doses of allopurinol could change the severity of the redox state, effectively creating a somewhat controllable model of oxidative stress. We made use of this model to investigate plasma ceramides in broiler chickens. Changes in plasma ceramide concentrations were first determined during the growth phase and in response to different treatments.

Measurement of plasma ceramides in control birds over the course of the 4-week sampling period revealed an overall significant decline in plasma ceramides, although between week 5 and 6 some species significantly increased (C22:0 and C24:0) whereas others (C16:0, C18:0 and C20:0) remained depressed. These results did not support our initial prediction, as plasma ceramide concentrations decreased with age in broiler chicks as has been observed in other species (Lightle et al., 2000). These observations may result from a decrease in the growth rate observed at 5 weeks of age in broiler chickens as has been previously described (Kuenzel and Kuenzel, 1977). Schols et al. (Schols et al., 1996) suggested that an increase in basal metabolic rate in humans was associated with an increase in the inflammatory state as indicated by the increase in inflammatory markers. Hence, the downturn in metabolic rate and decrease in oxygen (O_2_) consumption was suggested to parallel the decrease in plasma ceramides concentrations. If the downtrend was prolonged past week 5 of age as measured by Kuenzel et al. (Kuenzel and Kuenzel, 1977) this would support the negative correlation (*r = -0.57*) observed between the increase in body weight and the decrease in plasma ceramide concentrations.

The liver is the primary organ for free fatty acid synthesis in birds (Whittow, 1999). Ceramide synthesis occurs via the addition of a fatty acid moiety to a sphingosine back bone via ceramide synthase (Bikman and Summers, 2011). In this study, non-esterified fatty acids concentrations were relatively constant until a significant decline (*p* < 0.05) was observed between weeks 5 and 6, with this decrease reciprocally paralleling the increase in plasma ceramides during this same period. Watt et al. (Watt et al., 2012) demonstrated that ceramide synthesis was dependent on substrate availability rather than liver serinepalmitoyl transferase (SPT) activity. These results suggest that a fraction of available free fatty acids may have been shuttled towards ceramide synthesis, while free fatty acids levels were not restored to their original levels. As ceramides are markers of oxidative stress and positively correlated with inflammation characterized by ROS production (Birch and Schreiber, 1986; Cutler et al., 2002), this scenario could also account for the increase (*p* < 0.055) in PUA, a potent antioxidant, during that period.

The induction of an inflammatory state by allopurinol was evident by the significant decreases in plasma uric acid. However, plasma ceramides in the allopurinol fed groups did not increase as predicted, rather there was a significant decrease in most species measured. The inflammatory state is associated with an increase in protein synthesis as observed in rats, including various inflammatory proteins such as interleukins and their counterparts (anti-inflammatory and antioxidants proteins) (Schreiber et al., 1982; Vary and Kimball, 1992; Knight et al., 2005). This increase in liver protein synthesis coupled with a decrease in feed intake caused by allopurinol may have contributed to the decrease in plasma ceramides concentrations. Comparably, serine amino acids, which are required for ceramide synthesis, could be redirected towards protein synthesis as well (Vary and Kimball, 1992; Knight et al., 2005). Schreiber et al. (Schreiber et al., 1982) demonstrated that a labeled pool of liver leucine in rats was incorporated into plasma proteins, for up to 3 days after the challenge as result of inducing inflammation.

NEFA were significantly elevated in all treatments compared to controls. This increase in NEFA may be a result of the inclusion of CLA, BRB and allopurinol in the diet. BRB has been suggested to inhibit adipogenesis in mammals (Choi et al., 2006; Hu and Davies, 2010), which could explain the observed increase in NEFA. Conjugated linoleic acid is known to increase the rate of lipolysis in adipose tissues, which would explain the increase in circulating fatty acids (Chung et al., 2005). Although similar results were observed in Moulard ducks (hybrid of Muscovy and Pekin ducks) (Fesler and Peterson, 2013); a decrease in total hepatic NEFA (which would suggest a decrease in serum NEFA) was observed in chickens fed CLA. Previous studies in ApoE KO mice supplemented with 10 μM of allopurinol for 4 weeks revealed a numerical increase in serum NEFA, which was suggested to be the result of allopurinol inhibiting the uptake of low density lipoprotein (LDL) into macrophages. In a second study the authors increased the expression of xanthine oxidoreductase in cell cultures which increased LDL uptake in macrophage cells resulting in foam cell formation (Kushiyama et al., 2012). The combined synergistic effect of BRB + ALLO would explain the difference (*p = 0.0504*) in NEFA observed between the BRB and BRB + ALLO groups. However, only a numerical increase in NEFA was noted in the CLA + ALLO group compared to CLA.

Both plasma ceramides C16 and C18 in controls and treatments were the least changed compared to the other ceramide species. There was a significant linear decrease in C16 and C18 in controls, however, there was a numerical decrease in C16 and C18 concentrations when challenged with allopurinol. Ceramides C16 and C18 have been linked to insulin resistance (Grösch et al., 2012). Turpin et al. (Turpin et al., 2014) demonstrated that an increase in ceramide C16 further aggravated the insulin resistance state in mice. In a study where Ceramide synthase 6 (CerS6) knockout mice were used to investigate the absence of ceramide C16, the authors observed a decrease in the ceramide C16 coupled with an improvement in insulin sensitivity and an increase energy expenditure. In addition, Ceramide synthase (CerS) 6 and 5 (producers of Cer C16:0 and C18:0) are equally expressed in a greater number of tissues than Cers 2 and 4 (producers of Cer C20:0- C24:0), which are mostly expressed in liver and kidneys (Andrieu-Abadie et al., 2001). If this pattern of expression is similar in birds, this might help explain the decrease in plasma ceramides C20 to C24 in the broiler chickens fed allopurinol. However, this explanation does not explain the decrease in plasma C16 with allopurinol.

Measurement of ceramide in broiler chickens as markers of oxidative stress is feasible as they have already been correlated with oxidative stress and ROS production in other species (Cutler et al., 2002; Cutler et al., 2004). In addition, treatments for some ceramide specific associated diseases have already been devised. In human patients with amyotrophic lateral sclerosis (ALS), the authors observed an increase and accumulation of ceramide C16 in neurons which resulted in the death of the neurons. They also demonstrated that treatment with the antibiotic myriocin, an inhibitor of SPT, resulted in a decrease in ceramide C16 accumulation in cells (Cutler et al., 2004). A provisional analysis of ceramides in growing broiler chickens, revealed a decline towards the end of the growth phase (weeks 2–∼8 of age). Under conditions of oxidative stress, most species of ceramides are decreased, however, this may be an indirect consequence of inducing an inflammatory state. The administration of allopurinol specifically targets the liver which in birds, is the primary organ for fatty acids synthesis. For this reason, ceramide production might have been unwittingly affected by the addition of allopurinol. Alternative methods to induce an oxidative stress state challenge should be investigated to corroborate the findings of this study.

## Data Availability

The data analyzed in this study was obtained from Dr. J. W. McFadden. Requests to access these datasets should be directed to Dr. McFadden (McFadden@cornell.edu).
